# Effects of OxyR regulator on oxidative stress, Apx toxin secretion and virulence of *Actinobacillus pleuropneumoniae*

**DOI:** 10.3389/fcimb.2023.1324760

**Published:** 2024-01-10

**Authors:** Fangfang Guo, Rong Quan, Yifang Cui, Xiaoya Cao, Tong Wen, Fuzhou Xu

**Affiliations:** ^1^ Beijing Key Laboratory for Prevention and Control of Infectious Diseases in Livestock and Poultry, Institute of Animal Husbandry and Veterinary Medicine, Beijing Academy of Agriculture and Forestry Sciences, Beijing, China; ^2^ Department of Biology Science and Technology, Baotou Teacher’s College, Baotou, China

**Keywords:** *Actinobacillus pleuropneumoniae*, oxyR gene, Apx toxins, oxidative stress, virulence

## Abstract

**Introduction:**

*Actinobacillus pleuropneumoniae*, the causative agent of porcine pleuropneumonia, poses a significant threat to global swine populations due to its high prevalence, mortality rates, and substantial economic ramifications. Understanding the pathogen's defense mechanisms against host-produced reactive oxygen species is crucial for its survival, with OxyR, a conserved bacterial transcription factor, being pivotal in oxidative stress response.

**Methods:**

This study investigated the presence and role of OxyR in *A. pleuropneumoniae* serovar 1-12 reference strains. Transcriptomic analysis was conducted on an oxyR disruption mutant to delineate the biological activities influenced by OxyR. Additionally, specific assays were employed to assess urease activity, catalase expression, ApxI toxin secretion, as well as adhesion and invasion abilities of the oxyR disruption mutant on porcine 3D4/21 and PT cells. A mice challenge experiment was also conducted to evaluate the impact of oxyR inactivation on *A. pleuropneumoniae* virulence.

**Results:**

OxyR was identified as a conserved regulator present in *A. pleuropneumoniae* serovar 1-12 reference strains. Transcriptomic analysis revealed the involvement of OxyR in multiple biological activities. The oxyR disruption resulted in decreased urease activity, elevated catalase expression, enhanced ApxI toxin secretion—attributed to OxyR binding to the apxIBD promoter—and reduced adhesion and invasion abilities on porcine cells. Furthermore, inactivation of oxyR reduced the virulence of *A. pleuropneumoniae* in a mice challenge experiment.

**Discussion:**

The findings highlight the pivotal role of OxyR in influencing the virulence mechanisms of *A. pleuropneumoniae*. The observed effects on various biological activities underscore OxyR as an essential factor contributing to the pathogenicity of this bacterium.

## Graphical Abstract

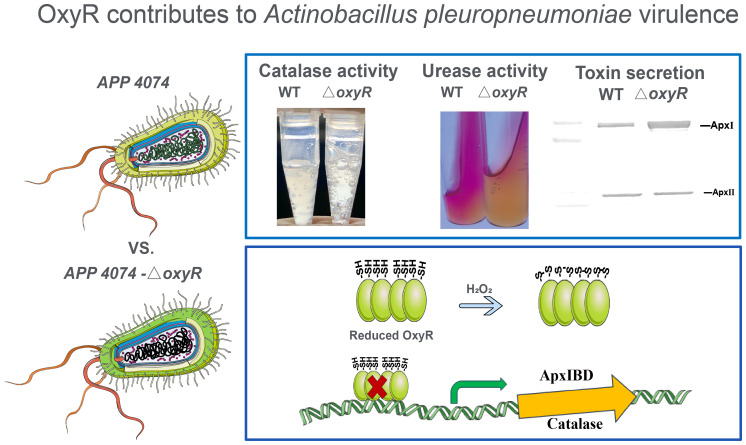

## 1 Introduction

*Actinobacillus pleuropneumoniae* is a gram-negative bacterium that causes porcine *pleuropneumonia*, which is characterized by acute hemorrhagic fibrous porcine *pleuropneumonia* with respiratory distress and results in significant economic losses on pig farms worldwide (Bossé et al., 2002; Sassu et al., 2018). The pathogenesis of *A. pleuropneumoniae* is extremely complex and involves multiple virulence determinants, including capsular polysaccharide, lipopolysaccharide, Apx toxins, outer membrane protein, and type IV fimbriae, etc. These virulence factors are required for colonization, nutrient acquisition, and evasion of host defense mechanisms (Bossé et al., 2002). The Apx toxins belong to the repeats-in-toxin (RTX) family and are the most important virulence factors influencing the pathogenesis of *A. pleuropneumoniae*. ApxI exhibits strong hemorrhagic and cytotoxic effects, which can cause cell damage and apoptosis in porcine alveolar macrophages (Chien et al., 2009). The detailed mechanism underlying the transcriptional regulation of Apx toxins remains unclear.

Bacteria encounter various harmful reactive oxygen species (ROS) in the normal metabolic process and living environment, such as superoxide anion free radicals (O^2-^), hydrogen peroxide (H_2_O_2_), and hydroxyl free radicals (HO˙) (Imlay, 2013). These substances can be produced via bacterial metabolism or by host cells to resist bacterial infection (Khomich et al., 2018). *A. pleuropneumoniae* has an effective regulatory mechanism for resisting oxygen stress; it can survive in alveolar macrophages for more than 90 min (Bossé et al., 2002). SodC is a copper-zinc superoxide dismutase protein that protects *A. pleuropneumoniae* from the superoxide generated by host cells (Langford et al., 1996). However, the pathogenicity of a *sodC* mutant of *A. pleuropneumoniae* remains unchanged (Sheehan et al., 2000). Moreover, little is known about the antioxidant mechanism used in *A. pleuropneumoniae*.

Previous studies have focused on characterizing anti-oxidative stress regulatory genes in several pathogens (Xia et al., 2017a; Mendis et al., 2018; Naito et al., 2021). OxyR, which was first identified in *Salmonella enterica* serovar Typhimurium (Christman et al., 1985), is one of the most widely studied oxidative stress regulators in bacteria (Imlay, 2015). As a DNA-binding protein of the LysR-family, OxyR responds to H_2_O_2_ stress by forming an intramolecular disulfide bond between two conserved cysteine residues (Zheng et al., 1998). It contains four different functional domains, namely DNA binding, tetramerization, H_2_O_2_-sensing, and transactivation domains. Wang et al. identified amino acid residues that are essential for each function, and these amino acid residues are usually conserved in OxyR from other bacterial species (Wang et al., 2006). In pathogenic bacteria, mutations in the *oxyR* gene reduce bacterial tolerance to oxidative stress, consequently decreasing the virulence or the ability of the pathogenic bacteria to infect. (Parti et al., 2013; Zhang et al., 2018). OxyR also regulates the *dps* gene, which is involved in iron storage. Dps protein can store ferrous iron in the form of Fe(OH)_3_, thereby reducing the concentration of ferrous iron and H_2_O_2_ generated by the Fenton reaction (Xia et al., 2017; Niu et al., 2020).

In this study, we identified a regulatory mechanism based on the LysR family transcriptional regulator OxyR (APPSER1_07700) in *A. pleuropneumoniae* strain 4074. Herein, an *oxyR* disruption mutant was constructed to characterize phenotypes associated with the putative oxidative stress response. Transcriptomic analysis revealed that OxyR influences the expression levels of 216 genes, indicating a broader regulatory role for OxyR in overcoming oxidative stress. Our experiments demonstrated that in *A. pleuropneumoniae*, OxyR regulates oxidative stress responses to enhance resistance against ROS and represses urease through an unidentified pathway. Moreover, OxyR negatively regulates expression of the ApxI toxin by directly binding to *apxIBD* putative promoter regions. In addition, mouse challenge experiment demonstrated that the virulence of the *oxyR* disruption mutant was attenuated. This is the first study to demonstrate that OxyR regulates toxin production and other virulence traits in *A. pleuropneumoniae* and closely related organisms.

## 2 Materials and methods

### 2.1 Bacterial strains and culture conditions

The bacterial strains, primers, and plasmids used in this study are listed in **Supplementary Tables S1** and **S2**. *A. pleuropneumoniae* reference strain 4074 (serovar 1) was routinely grown in tryptic soy broth (TSB) or agar (Difco, BD, Franklin Lakes, NJ, USA), with both media supplemented with 10 μg/mL of nicotinamide adenine dinucleotide (NAD; Sigma), at 37°C unless mentioned otherwise. For the selection of *A. pleuropneumoniae* transformants, chloramphenicol (5 μg/mL) was added. When culturing different *Escherichia coli* strains, appropriate antibiotics were added to Luria-Bertani (LB) broth or agar. For cultivation of *E. coli* β2155, diaminopimelic acid (DAP; 50 μg/mL) (Sigma-Aldrich, St. Louis, MO, USA) was added to LB medium (Li et al., 2018).

### 2.2 Construction of the △*oxyR* mutant strain

To construct the *A. pleuropneumoniae* 4074 mutant strain with specific gene inactivation, the ClosTron technique, a group II intron-based TargeTron system (Heap et al., 2007), modified for *Clostridium* genetics was used in this study. The pCD4C *LtrA* and *LtrB* genes were cloned directly into the pEMOC2 plasmid using the seamless cloning method to build the Targetron plasmid pEA. The insertion site in the antisense strand at position 138|139s in *oxyR* ORF was chosen for TargeTron modification using a computer algorithm (TargeTronTM, Sigma-Aldrich) as described previously (Perutka et al., 2004). PCR was used to modify intron sequences (EBS1, EBS2, and δ) using three pairs of partially point mutation primers *oxyR*LtrB 1F/R, *oxyR*LtrB 2F/R, and *oxyR*LtrB 3F/R (**Supplementary Table S2**).

The mutant △*oxyR* was then constructed using the plasmid pEAΔ*oxyR*, as described previously (Liu et al., 2015). The mutant strains were screened using *oxyR*YF/R primers. The resultant mutant strain △*oxyR* was examined using sodium dodecyl sulfate–polyacrylamide gel electrophoresis (SDS-PAGE) and Western blot with anti-*oxyR* rabbit polyclonal antisera (dilution 1:1000), following the standard procedures.

### 2.3 Production of recombinant OxyR and generation of polyclonal antisera

The *oxyR* gene was cloned by PCR from *A. pleuropneumoniae* 4074 using the primers P28*oxyR*-F/R, which contained *Nde*I and *Xho*I sites, respectively. The amplified fragment was ligated to pMD18-T for sequencing, digested with *Nde*I and *Xho*I, and ligated to digested pET28a (+) to construct the plasmid pET28a-*oxyR*. The pET28a-*oxyR* plasmid was transformed into *E. coli* BL21 (DE3) for expression. After cloning and successful expression, the rOxyR was purified by the AKTA Purifier 100 System (GE Healthcare, Bucks, United Kingdom) using a HisTrap FF affinity chromatography column.

Two male experimental Japanese big-eared white rabbits (about 2.5 kg, 8 weeks old) labeled as rabbit 1 and rabbit 2 were initially raised for a week. For immunization, rOxyR (1mg) antigen was mixed with Freund’s complete adjuvant (Sigma, St. Louis, Mo, USA) and injected subcutaneously into rabbits 1 and 2. After 12 days, a second immunization was administered using half the antigen volume and Freund’s incomplete adjuvant. Subsequent immunizations occurred every two weeks with the same antigen-adjuvant mixture. Blood (1 mL) was collected after each immunization for anti- rOxyR serum preparation, isolated a week after the final immunizations upon antibody detection. OxyR antiserum were purified by antigen affinity purification using the rOxyR.

### 2.4 RNA-Seq

Three independent cultures of *A. pleuropneumoniae* 4074 wild type (WT) and △*oxyR* were cultured in TSB for 7 h for RNA-Seq. Total RNA of each strain was extracted using the TRIzol reagent (Invitrogen, Waltham, MA, USA) according to the manufacturer’s instructions. Subsequently, DNaseI (Takara Bio, Kusatsu, Japan) was used to remove DNA from the extracted total RNA. Large ribosomal RNA was depleted from the total RNA using the RiboMinus Bacteria Module (Invitrogen). Single-end index libraries were constructed according to the manufacturer’s protocol (NEB Next^®^ Ultra™ RNA Library Prep Kit for Illumina^®^, San Diego, CA, USA). The library was sequenced on an Illumina HiSeq 4000 platform at Majorbio (Shanghai, China), and the data were analyzed on the Majorbio Cloud Platform 1. Clean sequence reads were mapped to the *A. pleuropneumoniae* chromosome using Bowtie. mRNA expression levels and transcripts per million (TPM) reads were calculated for each gene using RSEM (RNA-Seq by Expectation-Maximization). The TPM distribution density and correlation between the two groups were also analyzed to validate the reliability of the sequencing data.

Differentially expressed genes (DEGs) between WT and △*oxyR* strains were identified using DESeq2 with a Benjamini-Hochberg false discovery rate (FDR) used to determine the threshold p value for assigning statistical significance after multiple tests. A fold change value ≥ 2 and an FDR ≤ 0.005 were used as thresholds for identifying significant differential expression.

### 2.5 H_2_O_2_ growth assay

The growth rates of *A. pleuropneumoniae* were measured in TSB+NAD broth using a Bioscreen C Analyzer (Oy Growth Curves Ab Ltd, Helsinki, Finland). The concentration of an overnight culture of the strains in TSB+NAD broth was adjusted to be OD_600_ = 1.0. The adjusted cultures were then diluted 100-fold, and 100 μL of bacteria were inoculated per well in a 100-well plate. The cultures were grown at 37°C for 24 h, and the OD_600_ measurements were taken every 1 h with shaking. The growth curve of each strain was drawn using the OD_600_ measurements. The final concentration of H_2_O_2_ in the culture was 3 mM. The final concentration of FeCl_3_ in the culture was 5 μM. The growth rates were measured three times in duplicate.

### 2.6 Urease and catalase activity assay

To quantify the urease activity, bacteria cultured in the TSB+NAD broth were centrifuged at 10,000 ×*g* for 3 min after incubating on a shaker at 37°C for 6 h. The cell pellets were washed twice and suspended in 50 mM sodium phosphate buffer (pH 7.6). Urease and catalase activities were determined following the instructions of the QuantiChrom Urease Assay Kit (BioAssay Systems, Hayward, CA, USA) and Biochemical assay kit (Abbkine, Wuhan, China). All analyses were conducted in triplicates, and the data calculated from three independent experiments were analyzed using the student’s t-test.

### 2.7 Apx toxin secretion assay

To determine the level of ApxI and ApxII toxins secretion in supernatants of *A. pleuropneumoniae*, cultures was evaluated as described previously (Guo et al., 2021). Briefly, all strains were cultured in *pleuropneumonia*-like organism (PPLO) broth (Difco, BD) supplemented with 10 μg/mL NAD. The overnight cultures were inoculated into fresh PPLO+NAD medium with the addition of CaCl_2_ at a final concentration of 25 mM. After shaking incubation at 37°C for 6 h, the values of all the cultures were adjusted to be OD_600_ = 1.0. Subsequently, 10 mL of the adjusted culture of each strain was harvested by centrifuging at 10,000 ×*g* for 10 min at 4°C. The supernatants were concentrated 20-fold using an Amicon Ultra-15 10 kDa filtration centrifugal tube (Millipore, Burlington, MA, USA). The Apx toxins in the concentrated supernatants of each strain were examined using SDS-PAGE and Western blot with the anti-rApxI and anti-rApxII rabbit polyclonal antisera (dilution 1:200), following the standard procedures, as described previously (Guo et al., 2021).

### 2.8 Electrophoretic mobility shift assay

Electrophoretic mobility shift assay (EMSA) was performed using a Lightshift Chemiluminescent EMSA Kit (Thermo Fisher Scientific, Waltham, MA, USA). The DNA probes were amplified by PCR using the primers (**Supplementary Table S2**) and labelled using the Biotin 3’-end labeling kit (Thermo Fisher Scientific). The probes were then mixed with the appropriate amount of rOxyR protein and 0.5 μl of poly [d(I-C)] distributed in binding buffer to a final volume of 10 μl. The mixture was incubated at 25°C for 30 min and mixed with 5 mL 5 × loading buffer with bromophenol blue. Protein-bound and free DNA were separated by electrophoresis on non-denaturing 5% polyacrylamide gels in 0.5 × TBE running buffer (0.5 × TBE buffer contains 5.4 g of Tris base, 2.75 g of boric acid, and 2 ml of 0.5 M pH 8.0 EDTA per liter of deionized water) running and transferred from the gels onto a nylon membrane by electroblotting. After baking for 10 min at 80°C, the membrane was exposed to UV radiation at 256-nm for 10 min to cross-link the DNA fragments and the membrane. Chemiluminescence detection was performed according to the manufacturer’s instructions, and the results were observed using a chemiluminescent imager (Tanon, Bio-Equip, Shanghai, China).

### 2.9 Adhesion and invasion assay

*A. pleuropneumoniae* was cultured to logarithmic growth phase in TSB medium at 37°C and diluted to 1 × 10^8^ CFU/mL. Porcine alveolar macrophage 3D4/21 and porcine tonsil epithelial immortalized cell PT (1 × 10^6^) were washed with RPMI 1640 three times. One mL of the bacterial suspension was added with a multiplicity of infection (MOI) of 100. The mixture was cultured in 5% CO_2_ at 37°C for 2 h and was washed five times with phosphate-buffered saline (PBS). After adding 1 mL 0.025% (v/v) Triton X-100 for 10 min at 4°C, the lysates were counted using the plate counting method. Adhesion was calculated as the ratio of surface-adherent and intracellular bacteria relative to the total number of bacteria added initially (Duan et al., 2022). For invasion assays, gentamicin was also added to each well after washing with PBS and further cultured for 45 min. The cells were then lysed and diluted in a bacterial count gradient.

### 2.10 Mouse challenge experiment

This study was approved by the Institutional Review Board (IRB) of the Institute of Animal Husbandry and Veterinary Medicine, Beijing Academy of Agriculture and Forestry Sciences. Six-week-old BALB/c mice were purchased from the Beijing Vital River Laboratory Animal Technology Co., Ltd. (Beijing, China).

We equally divided 20 female BALB/c mice into *A. pleuropneumoniae* WT and Δ*oxyR* groups. Cultures of all strains were grown overnight at 37°C in TSB supplemented with NAD, followed by dilution to 1:1000. The diluted culture was incubated again until the OD_600_ reached 0.6, and washed three times with PBS. For the challenge group, bacteria at 1 × 10^7^ CFU per mouse were injected via the abdominal cavity, while an additional five control mice were injected with same volume of sterile PBS. The survival time of mice was observed for at least 48 h after bacterial injection. The mice were carefully observed, and those exhibiting severe signs of illness, such as dyspnea and depression, were humanely euthanized. The remaining mice were euthanized 48 hours after the inoculation.

To evaluate the colonization ability of *A. pleuropneumoniae* in the challenged mice, 12 female BALB/c mice were equally divided into *A. pleuropneumoniae* WT and Δ*oxyR* groups. Each group was intraperitoneally administered the same bacterial amounts of 5 × 10^5^ CFU per mouse. At 6 h post inoculation, the lung of each mouse was aseptically removed. Each lung sample (100 mg) was homogenized with 1 mL sterile PBS, and 100 μL of the lung homogenate were processed for determining the CFU counts. Recovered colonies of bacteria were counted to determine total CFU or CFU per gram organ.

### 2.11 Real-time quantitative reverse transcription PCR

Genes involved in oxygen stress, iron, and Apx toxin expression and secretion were selected for analysis at the transcriptional level by qRT-PCR. The primers used for qRT-PCR are listed in **Supplementary Table S2**. Total RNA was extracted from Ap1-wild type (WT) and △*oxyR* strains. The qRT-PCR was performed as described previously (Guo et al., 2021). The relative transcription level of each gene was determined by normalization to that of the 16S rRNA gene using the 2-△△Ct method.

### 2.12 Statistical analysis

Statistical analysis was performed using a one-way analysis of variance. Duncan’s multiple range test was used to compare the differences among the treatment groups. A p-value of < 0.05 was considered to indicate statistical significance.

## 3 Results

### 3.1 Bioinformatics analysis of the *oxyR* gene

**Figure 1:**
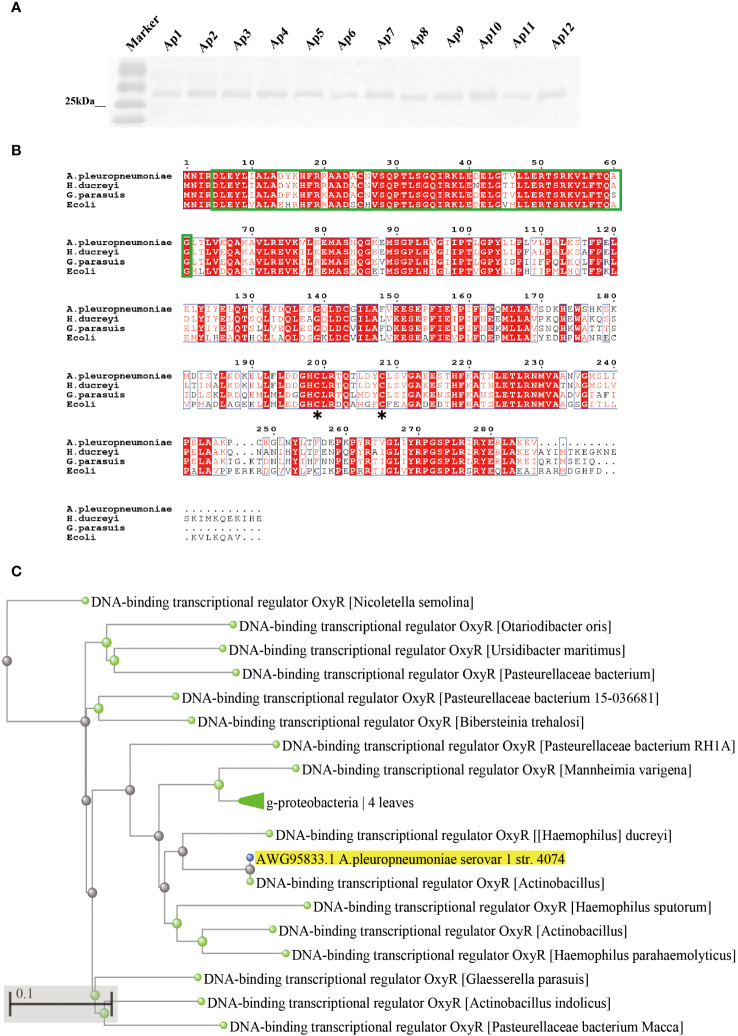
Prevalence and sequence alignment analysis of OxyR in *A. pleuropneumoniae*. **(A)** Analyses the distribution of OxyR in serovar 1-12 of *A. pleuropneumoniae* by Western blot. **(B)** Multiple alignments of OxyR transcriptional regulators from *A. pleuropneumoniae 4074* (AWG95833.1), *Haemophilus* (WP_071610708.1), *Haemophilus ducreyi* (WP_010944668.1) and *E. coli* (CAA36893.1). Conserved amino acid residues among these four proteins are highlighted by red, the proposed helix-turn-helix DNA-binding domain is marked by green, and the conserved reactive Cys-199 residues between *E. coli* and *4074* are marked by an asterisk. **(C)** Phylogenetic analysis of OxyR among different bacterial species. The asterisk (*) is used to mark the location of conserved cysteine residues in the OxyR protein.

The *oxyR* gene of *A. pleuropneumoniae* (APPSER1_07700) has a length of 901 bp and encodes a protein with 297 residues. The distribution of OxyR in *A. pleuropneumoniae* serovar 1-12 reference strains was investigated by Western blot with specific anti-OxyR antibodies. The results showed that the OxyR protein could be detected in all the strains (**Figure 1A**). Multiple sequence alignments between OxyR (AWG95833.1) and three other homologous proteins from *Glaesserella parasuis* (WP_071610708.1), *Haemophilus ducreyi* (WP_010944668.1), and *E. coli* (CAA36893.1) revealed that the OxyR of *A. pleuropneumoniae* has a relatively high amino acid sequence identity with *Glaesserella parasuis* (75%) and *Haemophilus ducreyi* (84%), but only 55% identity with *E. coli*. The sequence alignment results suggested that the LysR substrate binding domain (**Figure 1B**, green frame), and the LysR substrate binding domain amino acid residues Cys-199 have a high homology with the LysR family transcriptional regulator. We analyzed the OxyR protein by BLASTP search and confirmed 60 sequences with homology higher than 75%, and most of them are derived from pathogenic bacteria (**Figure 1C**).

### 3.2 Construction of the *oxyR* mutant

**Figure 2:**
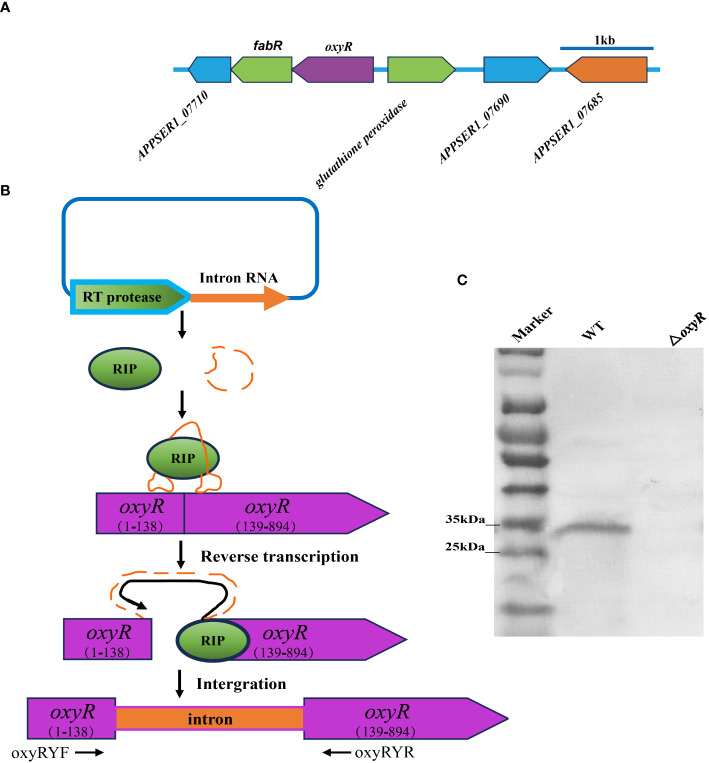
Intron-based mutagenesis of *oxyR* gene in *A. pleuropneumoniae* strain 4074. **(A)** The *oxyR* gene location in *A. pleuropneumoniae* genome. **(B)** The mature ribonucleoprotein complex (RNP) recognizes the target DNA by the principle of base complementary pairing. The intron sequence was reverse transcribed into cDNA and inserted into the target site by the reverse splicing process. **(C)** Identification of the *oxyR* gene mutant by Western blot using anti-OxyR specific antibody.

The flanking genes of *oxyR* in *A. pleuropneumoniae* encode the fabR regulator and the glutathione peroxidase, respectively (**Figure 2A**). To investigate the regulatory roles of OxyR, the ClosTron technique was used to construct an *oxyR* disruption mutant (**Figure 2B**). The successful construction of mutant strain △*oxyR* was determined by Western blot using the specific anti-OxyR antibodies (**Figure 2C**).

### 3.3 RNA-Seq analyses

**Figure 3:**
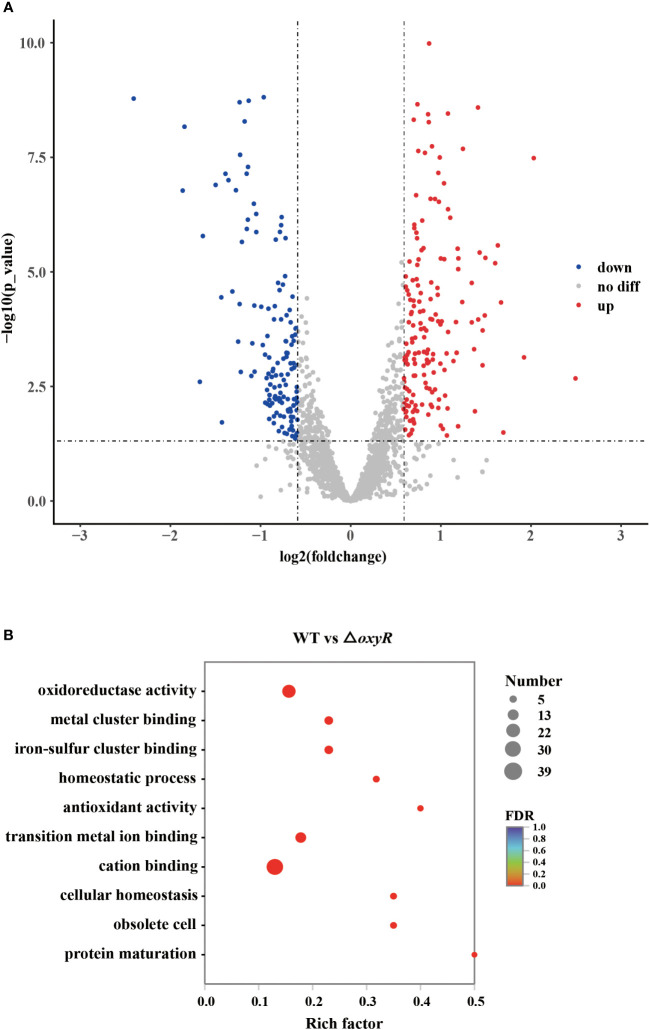
Transcriptomic analysis of the *oxyR* mutant. **(A)** Volcano plot showing gene expression. Red, green, and gray points represent upregulated, downregulated, and nonsignificant genes, respectively. **(B)** Pathways of differentially expressed genes analyzed by Gene Ontology.

To further investigate the globally regulatory roles of OxyR in *A. pleuropneumoniae*, we performed transcriptomic analyses using three biological replicates of the WT and △*oxyR* mutant strains. The results showed that on average there were 46,312,179 clean reads for the WT and 47,254,747 for the mutant. At least 94.3% of the DEGs were quantified. Detailed data on the DEGs are provided in **Supplementary Table S3**. These gene analyses identified 216 DEGs using statistical criteria (|log2 fold change| ≥ 1, FDR ≤ 0.005). Compared to the WT strain, 111 DEGs were upregulated and 81 DEGs were downregulated in the △*oxyR* mutant (**Figure 3A**).

Gene Ontology (GO) results showed that 26 genes were involved in oxidoreductase activity, 17 genes were involved in transition metal ion binding, 39 genes were involved in cation binding, and 6 genes were involved in antioxidant activity (**Figure 3B**). Most genes associated with oxidative stress, Apx toxin secretion, urea metabolic process, type I-F CRISPR and iron metabolism were regulated by OxyR (**Table 1**). The data obtained in this study suggested that OxyR plays a globally regulatory roles in the oxygen stress, toxin production, and virulence potential of *A. pleuropneumoniae*. Thus, the OxyR regulon identified by transcriptomic analysis is generally consistent with the phenotypic analysis described below.

**Table 1:** Differential expression genes (DEGs) between *A. pleuropneumoniae* WT and Δ*oxyR* strains.

Function	Product description	Gene ID	Log_2_R^a^
Oxidoreductase activity	hydrogenase 2 small subunit	APPSER1_RS07240	1.080376
	ribonucleotide-diphosphate reductase subunit beta	APPSER1_RS05455	1.341836
	ribonucleoside-diphosphate reductase subunit alpha	APPSER1_RS05450	1.429572
	molybdopterin-dependent oxidoreductase	APPSER1_RS09175	1.667295
	glycerol-3-phosphate dehydrogenase subunit GlpB	APPSER1_RS02035	3.75196
	anaerobic glycerol-3-phosphate dehydrogenase subunit C	APPSER1_RS02040	3.980692
	catalase	APPSER1_RS05435*	4.049482
	thiol peroxidase	APPSER1_RS08140*	-1.91597
	glutathione peroxidase	APPSER1_RS07695	-1.5886
	superoxide dismutase family protein	APPSER1_RS00020*	-1.49949
	DNA starvation/stationary phase protection protein	APPSER1_RS08165*	-1.4029
	heme anaerobic degradation radical SAM methyltransferase ChuW/HutW	APPSER1_RS08325	-1.31344
	thioredoxin	APPSER1_RS05915	-1.20739
	thioredoxin-disulfide reductase	APPSER1_RS00445	-1.15478
	aspartate-semialdehyde dehydrogenase	APPSER1_RS00025	-1.14156
	ISC system 2Fe-2S type ferredoxin	APPSER1_RS05140	-1.04767
	deferrochelatase/peroxidase EfeB	APPSER1_RS03575	-0.99253
Apx toxin activity	RTX family hemolysin ApxIA	APPSER1_RS07655	1.244011
	RTX family hemolysin	APPSER1_RS05270	1.035245
	RTX-II toxin-activating lysine-acyltransferase ApxIIC	APPSER1_RS05275	0.893181
Urea metabolic process	urease accessory protein UreG	APPSER1_RS08855	0.700443
	urease accessory protein UreE	APPSER1_RS08865	0.565418
	urease subunit beta	APPSER1_RS08880	0.768304
Type I-F CRISPR	type I-F CRISPR-associated endoribonuclease Cas6/Csy4	APPSER1_RS01100	1.486487
	type I-F CRISPR-associated protein Csy3	APPSER1_RS01105	1.236267
	type I-F CRISPR-associated protein Csy2	APPSER1_RS01110	1.0098
Ferric iron binding and regulator	heme utilization protein HutZ	APPSER1_RS05730	-4.26089
	ferric iron uptake transcriptional regulator	APPSER1_RS06640	-0.52987
	TonB-dependent siderophore receptor	APPSER1_RS10955	-2.37843
	TonB-dependent hemoglobin/transferrin/lactoferrin family receptor	APPSER1_RS05725	-1.44451

^a^WT/ΔoxyR. *Genes are associated with OxyR ChIP peaks.

### 3.4 OxyR regulates *A. pleuropneumoniae* growth and enzyme activity

**Figure 4:**
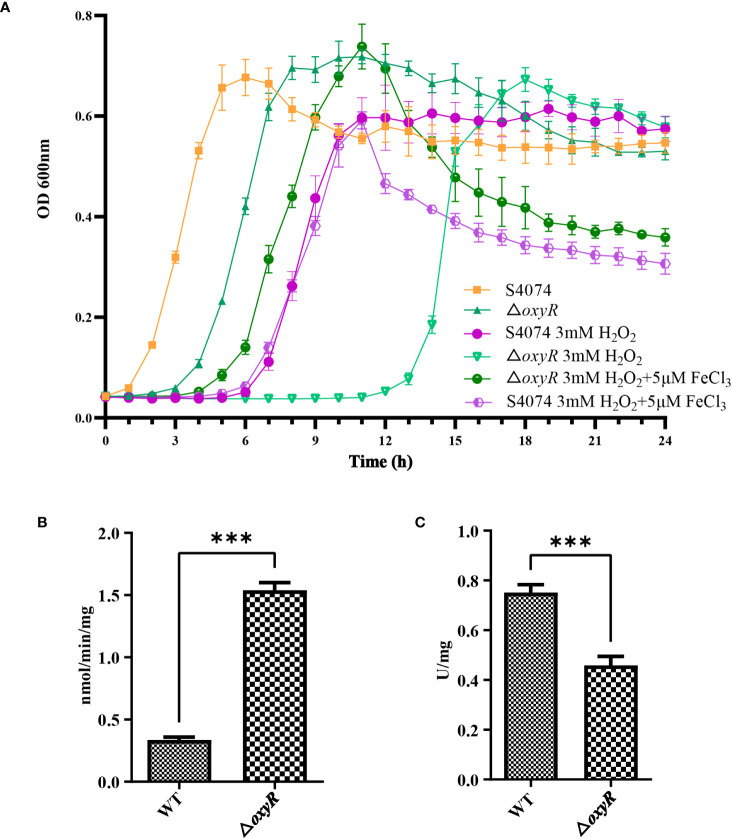
Effects of *oxyR* inactivation on the growth, catalase and urease activities of *A. pleuropneumoniae*. **(A)** Influence of H_2_O_2_ and FeCl_3_ on growth of WT *A. pleuropneumoniae* and the ΔoxyR strains. H_2_O_2_ was added to cultures of the wild-type and mutant strain to the final concentrations indicated. The cultures were kept on a shaker under the same growth conditions. **(B)** Catalase activity between the WT and *△oxyR* strains. **(C)** Urease activity between the WT and *△oxyR* strains. Data presented are the mean ± S.D. from three independent experiments performed in duplicate. ****p < 0.0001, ***p < 0.001, **p < 0.01, *p < 0.05.

The growth curve results showed that inactivation of *oxyR* impairs *A. pleuropneumoniae* growth in the TSB cultures with or without 3 mM H_2_O_2_ (**Figure 4A**). The significant growth lag was observed in the △*oxyR* strain with addition of 3 mM H_2_O_2_. Based on the downregulation of genes related to ferric iron binding and regulation in the transcriptome results (**Table 1**), we hypothesize that the △*oxyR* strain restricts iron acquisition under external hydrogen peroxide pressure. This limitation leads to a significant induction of catalase due to hydrogen peroxide, depleting iron ions and causing bacterial growth arrest or death. To validate this hypothesis, we conducted an experiment introducing 5 μM ferric chloride into the culture system. The results indicated increased tolerance of the △*oxyR* strain to 3 mM H_2_O_2_. Inactivation of *oxyR* also influences enzyme activities such as catalase and urease. Compared to the WT strain, the catalase activity in Δ*oxyR* increased three-fold (**Figure 4B**), while the urease activity of Δ*oxyR* decreased approximately 40% (**Figure 4C**). These results indicated that OxyR is involved in oxidative stress and other biological activities.

### 3.5 OxyR inhibited ApxI toxin secretion by binding to the promoter of *apxIBD*

**Figure 5:**
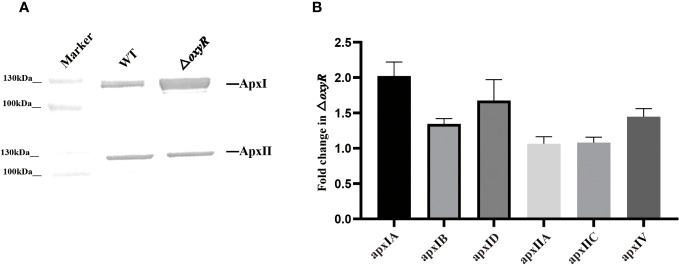
Secretion and expression levels of Apx toxins in WT and mutant strains. **(A)** Western blot analysis on secretion of ApxI and ApxII toxins in bacterial culture supernatants of WT and △*oxyR* strains. **(B)** Fold change in the expression level of apx genes in △*oxyR* by comparison with the WT strain.

To determine the regulatory roles of OxyR on Apx toxins secretion, ApxI and ApxII toxins in the supernatants of *A. pleuropneumoniae* WT and Δ*oxyR* cultures were detected by Western blot with specific anti-ApxI and anti-ApxII antibodies. The results showed that inactivation of *oxyR* dramatically promoted the secretion of ApxI while no influence on ApxII secretion (**Figure 5A**). Accordingly, compared with the WT strain, the transcriptional levels of apx*IA*, apx*IB*, and apx*ID* in △*oxyR* increased by 2, 1.4, and 1.7-fold, respectively, while no influence on apx*IIA* and apx*IIC* genes (**Figure 5B**). These results indicated that inactivation of *oxyR* inhibits the ApxI expression and secretion in *A. pleuropneumoniae*.

**Figure 6:**
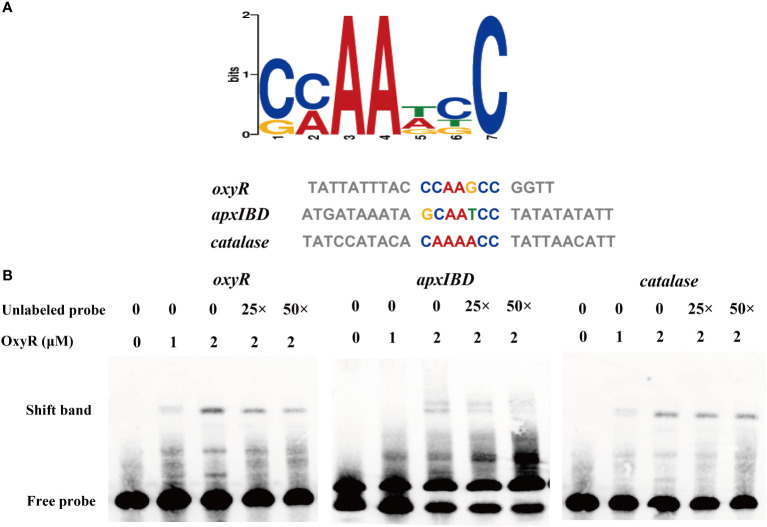
Identification of the potential OxyR binding motif. **(A)** Potential OxyR binding motif was identified by MEME. Representative sequences bound by OxyR in the EMSAs are listed below. The conserved sequence is shown in colors. **(B)** OxyR specifically binds to the *oxyR*, *apxIBD* and *catalase* promoters and the interactions between the protein and DNA were dissociated by unlabeled probe.

To identify genes directly regulated by the OxyR, we expressed the OxyR protein and prepared antibodies for the CHIP-seq assay. The results of the DNA binding sequences were obtained from the model-based analysis of ChIP-Seq (MACS) analysis software, and the gene corresponding to the nearest summit position (or midpoint if there is no summit position) of the peak was found, which was considered to have interrelated properties with the gene. The annotation results are shown in **Supplementary Table S4**. Notably, the potential regulatory targets of OxyR include genes involved in *catalase* and *oxyR*, and for the first time apx*IBD* toxin genes have also been associated. A MEME analysis on the ChIP-seq data revealed the *catalase, oxyR*, and apx*IBD* promoter’s potential binding site as 5′-CMAAWCC-3′, which was present in the probes used in the EMSAs (**Figure 6A**). To investigate the OxyR regulatory mechanism, rOxyR was purified and subjected to EMSAs. In the presence of multiple repeats, EMSAs revealed that OxyR clearly bound to the promoter region of the apx*IBD, catalase and oxyR* genes in a dose-dependent manner, and this binding was abolished by adding excess unlabeled competitor DNA (**Figure 6B**).

### 3.6 Inactivation of *oxyR* reduced virulence of *A. pleuropneumoniae*

**Figure 7:**
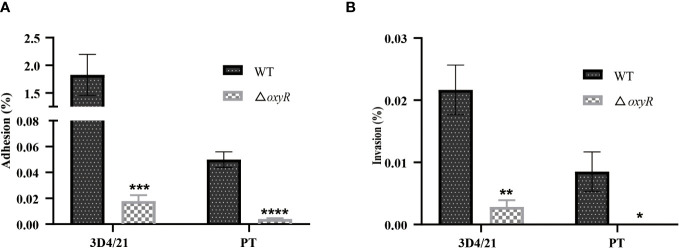
Cell adhesion and invasion assays. **(A)** Adhesion and **(B)** invasion of △*oxyR* strain. The strains were incubated with 3D4/21 and PT cells separately at the MOI of 100 for 2 h of incubation at 37°C. After washing out the unbound bacteria, the 3D4/21 and PT cells were lysed separately and the bacterial counts in the lysates were determined. Data are shown as mean ± SD (N = 6). ****p < 0.0001, ***p < 0.001, **p < 0.01, *p < 0.05.

The adhesion and invasion abilities of *A. pleuropneumoniae* WT and Δ*oxyR* to 3D4/21 and PT cells were investigated. Compared to the WT strain, the △*oxyR* mutant had a significant lower adhesion and invasion abilities both in 3D4/21 (**Figure 7A**) and PT cells (**Figure 7B**).

**Figure 8:**
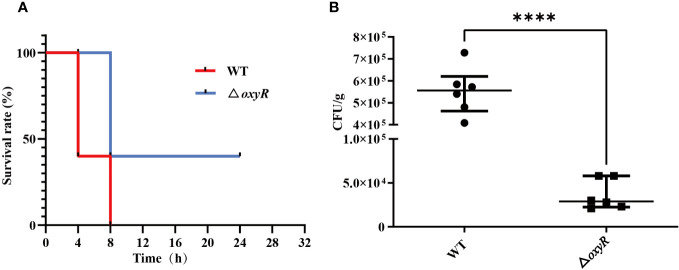
Virulence and colonization of the *A. pleuropneumoniae* △*oxyR* strains in a BALB/c mouse infection model. **(A)** Survival curves for *A. pleuropneumoniae* infected mice. Wild-type and △*oxyR* treated mice were monitored over a 32 hours period post infection. **(B)** Bacterial loads in the lungs. Mice were intraperitoneally inoculated with *A. pleuropneumoniae* strains. Lung samples were isolated to determine bacterial loads at 6 hours post infection. Significant differences are indicated by ****p < 0.0001, ***p < 0.001, **p < 0.01, *p < 0.05.

To investigate the influence of OxyR on virulence of *A. pleuropneumoniae in vivo*, the WT strain and △*oxyR* mutant were administered to mice via the intraperitoneal route. All the 10 mice in the WT group died within 12 h post challenge, while only 6 mice in the △*oxyR* group died until the end of the experiment (**Figure 8A**). The survival rate of mice in the △*oxyR* group was significantly higher than the WT group (p < 0.05). The bacterial loads of *A. pleuropneumoniae* WT and Δ*oxyR* in the lungs of challenged mice were also evaluated at a lower sublethal dose. While the WT strain showed high colonization levels. The bacterial loads in the lungs of the △*oxyR* group were significantly less than that of the WT strain at 6 h post challenge (**Figure 8B**). Collectively, these results indicated that OxyR plays an essential role in virulence of *A. pleuropneumoniae.*

## 4 Discussion

*A. pleuropneumoniae* is the causative agent of porcine *pleuropneumonia*, which can cause both acute lung infection and chronic asymptomatic infection by colonizing tonsils (Sassu et al., 2018). Under normal physiological conditions, the lung maintains an oxygen-rich environment, but lung infection and inflammation are characterized by hypoxic regions and high levels of ROS (Khomich et al., 2018). After phagocytosis, *A. pleuropneumoniae* can survive for up to 90 min in macrophages (Cruijsen et al., 1992). Therefore, it is very important to understand the antioxidant mechanism of *A. pleuropneumoniae*, because these mechanisms may play an important role in the colonization, growth, and virulence of bacteria.

The ability to alter gene expression in response to environmental stresses is essential for bacterial survival. OxyR is one of the most important transcriptional factors for anti-oxidative stress in many gram-negative bacteria, with the exception of *Deinococcus radiodurans* (Yin et al., 2010). It undergoes conformational changes after being induced by H_2_O_2_. OxyR positively regulates catalase and ahpC genes in γ-proteobacteria, such as *E. coli, Haemophilus, Pseudomonas, Salmonella*, and *Yersinia* (Loprasert et al., 2003). Currently, the most extensive and in-depth research on the transcriptional regulation mechanism of OxyR in *E. coli* is being conducted, which can regulate gene expression with non-antioxidant functions.

This research delved into the functions of OxyR in *A. pleuropneumoniae* pathogenesis and oxidative stress regulation. An analysis of reference strains of *A. pleuropneumoniae* revealed complete conservation of *oxyR* across all identified strains. Sequence analysis of *A. pleuropneumoniae oxyR* disclosed two conserved cysteine residues (Cys199 and Cys208), essential for oxidant-dependent OxyR activation. However, it only shares 55% sequence homology with *E. coli*. The Electrophoretic Mobility Shift Assay (EMSA) results demonstrated its direct binding to the promoter region of *apxIBD*, inhibiting ApxI toxin secretion. Moreover, hydrogen peroxide weakened the binding of OxyR protein to *apxIBD*, *oxyR*, *catalase*, and *APPSER1_RS1095* gene promoters (**Supplementary Figure**). Unlike most LysR regulators, our evidence suggests that in *A. pleuropneumoniae*, OxyR functions as a transcriptional repressor rather than a positive regulator of gene expression.

It is noteworthy that our transcriptome and catalase activity detection in whole bacteria highlighted an increase in catalase expression upon OxyR inactivation, potentially indicating OxyR’s role in catalase expression inhibition. However, the elevated catalase expression did not enhance *A. pleuropneumoniae’s* resistance to H_2_O_2_. This could be attributed to the absence of *oxyR* leading to diminished expression of iron metabolism-related genes like *fur*, *tonB* and *HutZ* (**Table 1**). This decrease in gene expression likely resulted in excessive catalase expression, depleting intracellular iron ions, thereby stalling or causing bacterial growth cessation or death. The absence of the *aphCF* operon, known for its role in scavenging microscale peroxide levels in the published *A. pleuropneumoniae* genome (Seaver and Imlay, 2001), alongside the reduced expression of other oxidoreductases (**Table 1**), exacerbated the sensitivity of *oxyR* mutation to H_2_O_2_.

Experimental results showed that the growth of *oxyR* mutants was significantly inhibited by 3 mM H_2_O_2_, as is the case for the vast majority of pathogenic bacteria with the *oxyR* mutation that are sensitive to H_2_O_2_. To validate our previous inference, additional ferric chloride was introduced into the H_2_O_2_-supplemented culture system, significantly alleviating the sensitivity of *oxyR* mutants to H_2_O_2_. This further corroborated the hypothesis that excessive expression of catalase leads to decreased intracellular iron content in bacteria and hampers their growth. The *oxyR* mutants exhibited deficiencies in the survival and growth of 3D4/21 and PT cells. Notably, *oxyR* mutants displayed notably lower rates of cell adhesion and invasion compared to the wild type, although transcriptomic data (**Table S4**) did not show significant changes in gene expression related to adhesion and biofilm formation. Though, there was a significant reduction in the expression levels of various virulence-associated or debilitation-related genes, such as *fur*, *tonB*, *HutZ*, *ybgC*, and *urease* (Dubuisson et al., 2005; Jacobsen et al., 2005; Shi et al., 2019; Liu et al., 2023), linked with iron metabolism, cell membrane composition, and response to oxygen stress. This has negatively impacted the *oxyR* mutant strain’s ability to adhere and invade host cells, hinting at OxyR’s role as a global transcriptional regulator. This observation suggests that the *oxyR* mutant is sensitive to reactive oxygen species within the host cell. This has been further validated in subsequent animal experiments, where the mutant strain exhibited significantly reduced colonization ability in the lungs of mice.

The mechanism by which *A. pleuropneumoniae* regulates oxidative stress response and secretion of Apx toxins remains unclear. Mutants lacking the two-component system *cpxA/cpxR*, *clpP*, and *arcA* gene in *A. pleuropneumoniae* display increased sensitivity to H_2_O_2_, suggesting their involvement in unconventional responses to oxidative stress (Buettner et al., 2008; Xie et al., 2013; Li et al., 2018; Li et al., 2019; Yan et al., 2020). Additionally, *malT* mutants exhibit slower growth rates and increased sensitivity to salt and serum (Lone et al., 2009). Transcription factors *sigmaE* and *H-NS* influence biofilm formation by modulating the expression of the *pga* operon (Bossé et al., 2010). The two-component system *QseB/QseC* regulates *pilM* transcription, whereby *pilM* mutants display reduced adhesion to Saint Jude porcine lung cells and diminished virulence in pigs (Liu et al., 2015). Although these genes showed no significant changes in transcriptome data, the downregulation of the key transcription factor, Ferric Uptake Regulator (Fur), which controls virulence, ROS defense, and iron absorption (Jacobsen et al., 2005; Troxell and Hassan, 2013), might contribute to the decreased virulence in oxyR mutant strains.

*A. pleuropneumoniae* can survive for an extended period within host alveolar macrophages, facing multiple stresses including low pH, reduced iron levels, ROS, and nitrogen species. This study demonstrates that under non-oxidative conditions, OxyR binds to the *apxIBD* promoter region but not directly to the *apxIA* promoter region. Upon activation by reactive oxygen species, OxyR releases the *apxIBD* promoter region, thereby promoting the secretion of ApxI toxin. ApxI exhibits strong hemolytic activity and cytotoxicity, causing lysis of porcine red blood cells and apoptosis in porcine alveolar macrophages (Hu et al., 2023). Surprisingly, the *oxyR* mutant displays increased catalase expression. While OxyR typically represses catalase, unlike its role in E. coli, in various organisms, it acts as both a repressor and an activator. For instance, in *Burkholderia pseudomallei*, *Shewanella oneidensis*, *Neisseria meningitidis*, *Neisseria gonorrhoeae*, *Corynebacterium diphtheriae*, and *Pseudomonas aeruginosa*, reduced OxyR inhibits catalase, while oxidized OxyR activates it (Tseng et al., 2003; Ieva et al., 2008; Heo et al., 2010; Jangiam et al., 2010; Kim and Holmes, 2012; Parti et al., 2013; Jiang et al., 2014). In these organisms, *oxyR* mutants exhibit higher basal catalase levels compared to wild-type cells.

Currently, direct evidence linking the urease operon to OxyR regulation is lacking, but the urease activity of the *oxyR* mutant is halved compared to the wild-type strain. In *mycobacteria*, urease inhibits host DNA repair, promotes lipid droplet formation, and contributes to bacterial survival (Liu et al., 2023). This might also contribute to the decreased virulence of the OxyR mutant. These findings suggest that *A. pleuropneumoniae* modulates *oxyR* gene expression to detect and respond to changes in H_2_O_2_ levels during infection, allowing for the over-secretion of ApxI toxin and catalase under stress conditions. Similar to *E. coli*, both the reduced and oxidized forms of OxyR inhibit its own expression by binding to the OxyR promoter, ensuring controlled OxyR levels unaffected by H_2_O_2_ pressure (Toledano et al., 1994). The undeniable link between oxidative stress and iron balance has drawn significant interest. OxyR decreases the intracellular iron pool by inducing Dps and Fur(Sen and Imlay, 2021). The virulence severely decreases in *fur* mutant of *A. pleuropneumoniae*, a phenomenon reconfirmed in *oxyR* mutant. The downregulation of *fur* expression might be one of the reasons for the observed virulence attenuation.

In conclusion, this study describes OxyR as an important virulence factor of *A. pleuropneumoniae*. Most importantly, this study provides novel insights into a mechanism of OxyR-dependent regulation of the oxidative stress response in *A. pleuropneumoniae*. Understanding of these unique pathogenic mechanisms is essential for tackling this important pathogen. In the future, we will further investigate the global expression network of the *A. pleuropneumoniae* OxyR.

## Data availability statement

The datasets presented in this study can be found in online repositories. The names of the repository/repositories and accession number(s) can be found in the article/**Supplementary Material**.

## Ethics statement

The animal study was approved by Institutional Review Board (IRB) of the Institute of Animal Husbandry and Veterinary Medicine, Beijing Academy of Agriculture and Forestry Sciences. The study was conducted in accordance with the local legislation and institutional requirements.

## Author contributions

FG: Formal analysis, Funding acquisition, Methodology, Project administration, Software, Visualization, Writing – original draft, Writing – review & editing, Data curation, Validation. RQ: Methodology, Resources, Writing – review & editing. YC: Data curation, Writing – review & editing. XC: Methodology, Writing – review & editing, Validation. TW: Visualization, Writing – review & editing, Conceptualization, Supervision. FX: Funding acquisition, Investigation, Supervision, Writing – review & editing, Project administration.

## References

[B1] Bossé J. T. Janson H. Sheehan B. J. Beddek A. J. Rycroft A. N. Kroll J. S.. (2002). *Actinobacillus pleuropneumoniae*: pathobiology and pathogenesis of infection. Microbes Infect. 4 (2), 225–235. doi: 10.1016/S1286-4579(01)01534-9 11880056

[B2] Bossé J. T. Sinha S. Li M.-S. O’Dwyer C. Nash J. H. Rycroft A. N.. (2010). Regulation of pga operon expression and biofilm formation in *Actinobacillus pleuropneumoniae* by σE and H-NS. J. Bacteriol. 192 (9), 2414–2423. doi: 10.1128/JB.01513-09 20207760 PMC2863473

[B3] Buettner F. F. Maas A. Gerlach G.-F. (2008). An *Actinobacillus pleuropneumoniae* arcA deletion mutant is attenuated and deficient in biofilm formation. Vet. Microbiol. 127 (1-2), 106–115. doi: 10.1016/j.vetmic.2007.08.005 17881160

[B4] Chien M.-S. Chan Y.-Y. Chen Z.-W. Wu C.-M. Liao J.-W. Chen T.-H.. (2009). *Actinobacillus pleuropneumoniae* serotype 10 derived ApxI induces apoptosis in porcine alveolar macrophages. Vet. Microbiol. 135 (3-4), 327–333. doi: 10.1016/j.vetmic.2008.09.071 19013727

[B5] Christman M. F. Morgan R. W. Jacobson F. S. Ames B. N. (1985). Positive control of a regulon for defenses against oxidative stress and some heat-shock proteins in Salmonella typhimurium. Cell 41 (3), 753–762. doi: 10.1016/S0092-8674(85)80056-8 2988786

[B6] Cruijsen T. Van Leengoed L. Dekker-Nooren T. Schoevers E. Verheijden J. (1992). Phagocytosis and killing of *Actinobacillus pleuropneumoniae* by alveolar macrophages and polymorphonuclear leukocytes isolated from pigs. Infect. Immun. 60 (11), 4867–4871. doi: 10.1128/iai.60.11.4867-4871.1992 1398997 PMC258242

[B7] Duan B. Peng W. Yan K. Liu F. Tang J. Yang F.. (2022). The QseB/QseC two-component system contributes to virulence of *Actinobacillus pleuropneumoniae* by downregulating apf gene cluster transcription. Anim. Dis. 2 (1), 2. doi: 10.1186/s44149-022-00036-w

[B8] Dubuisson J.-F. Vianney A. Hugouvieux-Cotte-Pattat N. Lazzaroni J. C. (2005). Tol-Pal proteins are critical cell envelope components of *Erwinia chrysanthemi* affecting cell morphology and virulence. Microbiology 151 (10), 3337–3347. doi: 10.1099/mic.0.28237-0 16207916

[B9] Guo F. Guo J. Cui Y. Cao X. Zhou H. Su X.. (2021). Exposure to sublethal ciprofloxacin induces resistance to ciprofloxacin and cross-antibiotics, and reduction of fitness, biofilm formation, and Apx toxin secretion in Actinobacillus *pleuropneumoniae* . Microb. Drug Resist. 27 (9), 1290–1300. doi: 10.1089/mdr.2020.0348 33739878

[B10] Heap J. T. Pennington O. J. Cartman S. T. Carter G. P. Minton N. P. (2007). The ClosTron: a universal gene knock-out system for the genus Clostridium. J. Microbiol. Methods 70 (3), 452–464. doi: 10.1016/j.mimet.2007.05.021 17658189

[B11] Heo Y. J. Chung I. Y. Cho W. J. Lee B. Y. Kim J. H. Choi K. H.. (2010). The major catalase gene (katA) of *Pseudomonas aeruginosa* PA14 is under both positive and negative control of the global transactivator OxyR in response to hydrogen peroxide. J. Bacteriol 192 (2), 381–390. doi: 10.1128/jb.00980-09 19933365 PMC2805318

[B12] Hu Y. Jiang C. Zhao Y. Cao H. Ren J. Zeng W.. (2023). TurboID screening of ApxI toxin interactants identifies host proteins involved in *Actinobacillus pleuropneumoniae*-induced apoptosis of immortalized porcine alveolar macrophages. Vet. Res. 54 (1), 62. doi: 10.1186/s13567-023-01194-6 37475032 PMC10360236

[B13] Ieva R. Roncarati D. Metruccio M. M. Seib K. L. Scarlato V. Delany I. (2008). OxyR tightly regulates catalase expression in *Neisseria meningitidis* through both repression and activation mechanisms. Mol. Microbiol. 70 (5), 1152–1165. doi: 10.1111/j.1365-2958.2008.06468.x 18990187

[B14] Imlay J. A. (2013). The molecular mechanisms and physiological consequences of oxidative stress: lessons from a model bacterium. Nat. Rev. Microbiol. 11 (7), 443–454. doi: 10.1038/nrmicro3032 23712352 PMC4018742

[B15] Imlay J. A. (2015). Transcription factors that defend bacteria against reactive oxygen species. Ann. Rev. Microbiol. 69, 93. doi: 10.1146/annurev-micro-091014-104322 26070785 PMC4618077

[B16] Jacobsen I. Gerstenberger J. Gruber A. D. Bossé J. T. Langford P. R. Hennig-Pauka I.. (2005). Deletion of the ferric uptake regulator Fur impairs the in *vitro* growth and virulence of *Actinobacillus pleuropneumoniae* . Infect. Immun. 73 (6), 3740–3744. doi: 10.1128/iai.73.6.3740-3744.2005 15908404 PMC1111875

[B17] Jangiam W. Loprasert S. Smith D. R. Tungpradabkul S. (2010). *Burkholderia pseudomallei* RpoS regulates OxyR and the katG-dpsA operon under conditions of oxidative stress. Microbiol. Immunol. 54 (7), 389–397. doi: 10.1111/j.1348-0421.2010.00230.x 20618685

[B18] Jiang Y. Dong Y. Luo Q. Li N. Wu G. Gao H. (2014). Protection from oxidative stress relies mainly on derepression of OxyR-dependent KatB and Dps in Shewanella oneidensis. J. Bacteriol 196 (2), 445–458. doi: 10.1128/jb.01077-13 24214945 PMC3911244

[B19] Khomich O. A. Kochetkov S. N. Bartosch B. Ivanov A. V. (2018). Redox biology of respiratory viral infections. Viruses 10 (8), 392. doi: 10.3390/v10080392 30049972 PMC6115776

[B20] Kim J. S. Holmes R. K. (2012). Characterization of OxyR as a negative transcriptional regulator that represses catalase production in *Corynebacterium diphtheriae* . PloS One 7 (3), e31709. doi: 10.1371/journal.pone.0031709 22438866 PMC3306370

[B21] Langford P. R. Loynds B. M. Kroll J. S. (1996). Cloning and molecular characterization of Cu, Zn superoxide dismutase from *Actinobacillus pleuropneumoniae* . Infect. Immun. 64 (12), 5035–5041. doi: 10.1128/iai.64.12.5035-5041.1996 8945543 PMC174485

[B22] Li Y. Cao S. Zhang L. Yuan J. Zhao Q. Wen Y.. (2019). A requirement of TolC1 for effective survival, colonization and pathogenicity of *Actinobacillus pleuropneumoniae* . Microbiol. Pathogen. 134, 103596. doi: 10.1016/j.micpath.2019.103596 31212036

[B23] Li H. Liu F. Peng W. Yan K. Zhao H. Liu T.. (2018). The CpxA/CpxR two-component system affects biofilm formation and virulence in *Actinobacillus pleuropneumoniae* . Front. Cell Infect. Microbiol. 8, 72. doi: 10.3389/fcimb.2018.00072 29662838 PMC5890194

[B24] Liu S. Guan L. Peng C. Cheng Y. Cheng H. Wang F.. (2023). *Mycobacterium tuberculosis* suppresses host DNA repair to boost its intracellular survival. Cell Host Microbe 31 (11), 1820–1836.e1810. doi: 10.1016/j.chom.2023.09.010 37848028

[B25] Liu J. Hu L. Xu Z. Tan C. Yuan F. Fu S.. (2015). *Actinobacillus pleuropneumoniae* two-component system QseB/QseC regulates the transcription of PilM, an important determinant of bacterial adherence and virulence. Vet. Microbiol. 177 (1-2), 184–192. doi: 10.1016/j.vetmic.2015.02.033 25796134

[B26] Lone A. G. Deslandes V. Nash J. H. Jacques M. MacInnes J. I. (2009). malT knockout mutation invokes a stringent type gene-expression profile in Actinobacillus pleuropneumoniae in bronchoalveolar fluid. BMC Microbiol. 9 (1), 1–15. doi: 10.1186/1471-2180-9-195 19751522 PMC2752462

[B27] Loprasert S. Sallabhan R. Whangsuk W. Mongkolsuk S. (2003). Compensatory increase in ahpC gene expression and its role in protecting *Burkholderia pseudomallei* against reactive nitrogen intermediates. Arch. Mirobiol. 180 (6), 498–502. doi: 10.1007/s00203-003-0621-9 14614594

[B28] Mendis N. Trigui H. Saad M. Tsang A. Faucher S. P. (2018). Deletion of *oxyR* in *Legionella pneumophila* causes growth defect on agar. Can. J. Microbiol. 64 (12), 1030–1041. doi: 10.1139/cjm-2018-0129 30212639

[B29] Naito M. Belvin B. R. Shoji M. Gui Q. Lewis J. P. (2021). Insertional inactivation of *Prevotella intermedia Oxyr* results in reduced survival with oxidative stress and in the presence of host cells. Microorganisms 9 (3), 551. doi: 10.3390/microorganisms9030551 33800047 PMC7999485

[B30] Niu W. Zhang Y. Liu J. Wen T. Miao T. Basit A.. (2020). OxyR controls magnetosome formation by regulating magnetosome island (MAI) genes, iron metabolism, and redox state. Free Radic. Biol. Med. 161, 272–282. doi: 10.1016/j.freeradbiomed.2020.10.015 33075503

[B31] Parti R. P. Horbay M. A. Liao M. Dillon J.-A. R. (2013). Regulation of minD by *oxyR* in Neisseria gonorrhoeae. Res. Microbiol. 164 (5), 406–415. doi: 10.1016/j.resmic.2013.02.002 23434849

[B32] Perutka J. Wang W. Goerlitz D. Lambowitz A. (2004). Use of computer-designed group II introns to disrupt escherichia coli DExH/D-box protein and DNA helicase genes. J. Mol. Biol. 336, 421–439. doi: 10.1016/j.jmb.2003.12.009 14757055

[B33] Sassu E. Bossé J. Tobias T. Gottschalk M. Langford P. Hennig-Pauka I. (2018). Update on Actinobacillus *pleuropneumoniae*—knowledge, gaps and challenges. Transbound Emerg. Dis. 65, 72–90. doi: 10.1111/tbed.12739 29083117

[B34] Seaver L. C. Imlay J. A. (2001). Alkyl hydroperoxide reductase is the primary scavenger of endogenous hydrogen peroxide in *Escherichia coli* . J. Bacteriol. 183 (24), 7173–7181. doi: 10.1128/JB.183.24.7173-7181.2001 11717276 PMC95566

[B35] Sen A. Imlay J. A. (2021). How microbes defend themselves from incoming hydrogen peroxide. Front. Immun. 1104. doi: 10.3389/fimmu.2021.667343 PMC8115020 33995399

[B36] Sheehan B. J. Langford P. R. Rycroft A. N. Kroll J. S. (2000). [Cu, Zn]-superoxide dismutase mutants of the swine pathogen *Actinobacillus pleuropneumoniae* are unattenuated in infections of the natural host. Infect. Immun. 68 (8), 4778–4781. doi: 10.1128/IAI.68.8.4778-4781.2000 10899887 PMC98436

[B37] Shi Y. J. Fang Q. J. Huang H. Q. Gong C. G. Hu Y. H. (2019). HutZ is required for biofilm formation and contributes to the pathogenicity of *Edwardsiella piscicida* . Vet. Res. 50 (1), 76. doi: 10.1186/s13567-019-0693-4 31578154 PMC6775658

[B38] Toledano M. B. Kullik I. Trinh F. Baird P. T. Schneider T. D. Storz G. (1994). Redox-dependent shift of OxyR-DNA contacts along an extended DNA-binding site: a mechanism for differential promoter selection. Cell 78 (5), 897–909. doi: 10.1016/S0092-8674(94)90702-1 8087856

[B39] Troxell B. Hassan H. M. (2013). Transcriptional regulation by Ferric Uptake Regulator (Fur) in pathogenic bacteria. Fronti. Cell. Infect. Microbiol. 3, 59. 10.3389/fcimb.2013.00059 PMC3788343 24106689

[B40] Tseng H. J. McEwan A. G. Apicella M. A. Jennings M. P. (2003). OxyR acts as a repressor of catalase expression in Neisseria gonorrhoeae. Infect Immun. 71 (1), 550–556. doi: 10.1128/IAI.71.1.550-556.2003 12496210 PMC143252

[B41] Wang X. Mukhopadhyay P. Wood M. J. Outten F. W. Opdyke J. A. Storz G. (2006). Mutational analysis to define an activating region on the redox-sensitive transcriptional regulator OxyR. J. Bacteriol. 188 (24), 8335–8342. doi: 10.1128/JB.01318-06 17012382 PMC1698235

[B42] Xia X. Larios-Valencia J. Liu Z. Xiang F. Kan B. Wang H.. (2017). OxyR-activated expression of Dps is important for *Vibrio cholerae* oxidative stress resistance and pathogenesis. PloS One 12 (2), e0171201. doi: 10.1371/journal.pone.0171201 28151956 PMC5289545

[B43] Xie F. Zhang Y. Li G. Zhou L. Liu S. Wang C. (2013). The ClpP protease is required for the stress tolerance and biofilm formation in Actinobacillus *pleuropneumoniae* . PloS One 8 (1), e53600. doi: 10.1371/journal.pone.0053600 23326465 PMC3543445

[B44] Yan K. Liu T. Duan B. Liu F. Cao M. Peng W.. (2020). The CpxAR two-component system contributes to growth, stress resistance, and virulence of *Actinobacillus pleuropneumoniae* by upregulating wecA transcription. Front. Microbiol. 11, 1026. doi: 10.3389/fmicb.2020.01026 32528444 PMC7255013

[B45] Yin L. Wang L. Lu H. Xu G. Chen H. Zhan H.. (2010). DRA0336, another OxyR homolog, involved in the antioxidation mechanisms in *Deinococcus radiodurans* . J. Microbiol. 48 (4), 473–479. doi: 10.1007/s12275-010-0043-8 20799089

[B46] Zhang Y. Chong X. Xia L. Lu R. Osei-Adjei G. Zhang Y.. (2018). OxyR positively and directly regulates Vi polysaccharide capsular antigen in Salmonella enterica serovar Typhi. Microb. Pathog. 124, 191–197. doi: 10.1016/j.micpath.2018.08.050 30145252

[B47] Zheng M. Åslund F. Storz G. (1998). Activation of the OxyR transcription factor by reversible disulfide bond formation. Science 279 (5357), 1718–1722. doi: 10.1126/science.279.5357.1718 9497290

