# Supplementary material for: Effects of OxyR regulator on oxidative stress, Apx toxin secretion and virulence of Actinobacillus pleuropneumoniae
Source: Front Cell Infect Microbiol. 2024 Jan 10;13:1324760. doi: 10.3389/fcimb.2023.1324760 (PMC10806198; doi:10.3389/fcimb.2023.1324760)
Supplement: Supplementary file 1 [file DataSheet_1.docx]

Supplementary Material

Effects of OxyR regulator on oxidative stress, Apx toxin secretion and virulence of Actinobacillus pleuropneumoniae

# Supplementary Tables

Supplementary Table S1. The bacterial strains and plasmids used in this study.

| Strains/plasmids | Characteristics | Source/Reference |
| --- | --- | --- |
| *A. pleuropneumoniae* | | |
| S4074 | *A. pleuropneumoniae* reference strain of serovar 1 | Dr P. Blackall |
| S1536 | *A. pleuropneumoniae* reference strain of serovar 2  CP031875 | China General Microbiological Culture Collection Center |
| S1421 | *A. pleuropneumoniae* reference strain of serovar 3  CP031874 | China General Microbiological Culture |
| M62 | *A. pleuropneumoniae* reference strain of serovar 4  CP031873 | China General Microbiological Culture |
| K17 | *A. pleuropneumoniae* reference strain of serovar 5a  CP069797 | China General Microbiological Culture |
| femo | *A. pleuropneumoniae* reference strain of serovar 6  CP069796 | China General Microbiological Culture |
| WF83 | *A. pleuropneumoniae* reference strain of serovar 7  CP031869 | China General Microbiological Culture |
| 405 | *A. pleuropneumoniae* reference strain of serovar 8  CP031866 | China General Microbiological Culture |
| CVJ13261 | *A. pleuropneumoniae* reference strain of serovar 9  CP031865 | China General Microbiological Culture |
| D13039 | *A. pleuropneumoniae* reference strain of serovar 10  CP031864 | China General Microbiological Culture |
| 56153 | *A. pleuropneumoniae* reference strain of serovar 11  CP031863 | China General Microbiological Culture |
| 8329 | *A. pleuropneumoniae* reference strain of serovar 12  CP031862 | China General Microbiological Culture |
| *△oxyR* | *A. pleuropneumoniae* S4074 *oxyR-*disruption mutant | This study |
| *E.coli* | | |
| DH5α | Cloning host for recombinant vector  CP031863 | Biomed |
| BL21 | The expression host for pET-28a and their derivative | Biomed |
| *β2155* | Transconjugation donor for constructing mutant strain | From Prof. |
| *PLASMID* | | |
| pEMOC2 | Transconjugation vector: ColE1 ori mob RP4 sacB, AmprCmr | Accession no. AJ868288 |
| pACD4C | Targetron plasmid, contains p15 ori T7 LtrB LtrA，Cmr | Lambowitz and Zimmerly (2011) |
| pEA | Targetron plasmid, pACD4C LtrB LtrA cloned into pEMOC2: ColE1 ori mob RP4 sacB, AmprCmr | This study |
| pEAΔ*oxyR* | oxyR intron product from pCE2--oxyR cloned into pEA (oxyR mutator plasmid); ColE1 ori mob RP4 sacB, AmprCmr | This study |
| pMD18-T | T-vector; Ampr | Takara |
| pMD18-T-*oxyR* | pMD18-T carrying *oxyR* genes | This study |
| pET-28a | Expression vector; Kanr | Novagen |
| pET-*oxyR* | pET-28a carrying *oxyR* gene | This study |

Comparative genomics of 26 complete circular genomes of 18 different serotypes of Actinobacillus pleuropneumoniae
