# Supplementary material for: Effects of OxyR regulator on oxidative stress, Apx toxin secretion and virulence of Actinobacillus pleuropneumoniae
Source: Front Cell Infect Microbiol. 2024 Jan 10;13:1324760. doi: 10.3389/fcimb.2023.1324760 (PMC10806198; doi:10.3389/fcimb.2023.1324760)
Supplement: Supplementary file 2 [file DataSheet_2.docx]

Supplementary Table S2. List of primers used in this study.

| Primers | Sequence（5‘-3’） | Source/Reference |
| --- | --- | --- |
| **For mutant construction** | | |
| LtrABF/R | gcaggtcgacgctagaaaaaTAATACGACTCACTATAGGGGAATTGTG | This study |
|  | accgcggtggcgtacccatcTCACTTGTGTTTATGAATCACGTGA |  |
| pEMOC2F/R | GATGGGTACGCCACCGCG | This study |
|  | TTTTTCTAGCGTCGACCTGCC |  |
| oxyRLtrB 1F/R | CTTATTAGGCACGGTAGTGCGCCCAGATAGGGTGTTAAG | This study |
|  | GCACTACCGTGCCTAATAAGGATAATTATAAGCTTTTTTCTAGCGTCGACCG |  |
| oxyRLtrB 2F/R | GATTCCTAATCGATAGAGGAAAGTGTCTGAAACCTCTAGTAC AGTAC | This study |
|  | CCTCTATCGATTAGGAATCGAAATTAGAAACTTGCGTTCAGTAAACACAAC GAATC |  |
| oxyRLtrB 3F/R | GGTAAGTTAAATACCGTGACTTATCTGTTATCACCACATTTGTACAATCTGTAGG | This study |
|  | GTCACGGTATTTAACTTACCTTTCTTTGTACTAGAGGTTTCAGACACTTTCC |  |
| oxyRYF/R | CAGCCGACACTAAGCGGTCA | This study |
|  | GCAGCGGTCCGGACATTTCT |  |
| oxyRSF/R | aaccgcacgcattctacac | This study |
|  | ccgcacttaattggttaaaagccg |  |
| **For Protein expression** | | |
| P28oxyR-F/R | GGAATTCCATATGgtgaatattagagatttagaatatttaatcgctttagcaga | This study |
|  | CCGCTCGAGctcatgaattttctcttgtttcataatcttggaaa |  |
| **For RT-PCR** | | |
| RS05435F | TCGTTGTCCGGTTCACAGTA | This study |
| RS05435R | TGCCGCATCACCGTTAATTT |  |
| \| UreaBF \| \| --- \| \| UreaB-R-RT 57 \| | TTGGTAGCAGGTTCGCAAGT | This study |
| \| UreaBR \| \| --- \| \| UreaB-R-RT 57 \| | GCGTGGATGCGAACATCAAA |  |
| UreaCF | ACGCAGTCAGTATGTAGCGA | This study |
| UreaCR | CGGTGCTTGATTGTGCCATA |  |
| 01095F | TACCGCATGGTTTGGCGATA | This study |
| 01095R | AGCGCTTCAAAGCGAACAAG |  |
| RS07400F | GTCAGCAGCTCGGTATCTCT | This study |
| RS07400R | CCAACGGCACAAGCAACTTT |  |
| TonBF | CCGGTTTCAACTACCGTCAC | This study |
| TonBR | GGCACAAGAGCAAATGCAAC |  |
| SODF | GCCGTGTTGCTTGGTATCTT | This study |
| SODR | TACTCCGCACTTACACGGTT |  |
| FurF | TTCGCTGATTTCACGCTGAC | This study |
| FurR | TCGGTTTGGCAACGGTTTAC |  |
| apxIAF | CCACTAACTGAGCGGCTAGA | This study |
| apxIAR | CGGCACTTTCAGCCTTACAA |  |
| apx1DF | AACATCCGCAACTCGGTCTA | This study |
| apx1DR | ACGGATTCTTCCAACGGACT |  |
| apx1BF | ATCGTTTGGCGTTTATCGCA | This study |
| apx1BR | GCTCTCGAATTCCGCTTGTT |  |
| ApxIICF | GTCCTTTACATAGCGAGCCTCA | This study |
| ApxIICR | ACGTATTGGGACAAATTGCTTGG |  |
| ApxIIAF | acagccgagacagaaaaagg | This study |
| ApxIIAR | tgtttggtgggtggcaattg |  |
| apxIVF | agagaaaacggcgactttcg | This study |
| apxIVR | aacggcgggcaattttagtc |  |
| 16srRNAF | ttcgatgcaacgcgaagaac | This study |
| 16srRNAR | tttcacaacacgagctgacg |  |
| **For EMSA** | | |
| oxyRpF/R | GAACGTACCAGCCGTAAGGTATTATTTACCCAAGCCGGTT | This study |
|  | AACCGGCTTGGGTAAATAATACCTTACGGCTGGTACGTTC |  |
| ApxIBDpF/R | ATGATAAATAGCAATCCTATATATATTAGGTGTGTAGGAT | This study |
|  | ATCCTACACACCTAATATATATAGGATTGCTATTTATCAT |  |
| 05430pF/R | ATAATAATATATCCATACACAAAACCTATTAACATTCCAA | This study |
|  | TTGGAATGTTAATAGGTTTTGTGTATGGATATATTATTAT |  |
