# Supplementary material for: Effects of OxyR regulator on oxidative stress, Apx toxin secretion and virulence of Actinobacillus pleuropneumoniae
Source: Front Cell Infect Microbiol. 2024 Jan 10;13:1324760. doi: 10.3389/fcimb.2023.1324760 (PMC10806198; doi:10.3389/fcimb.2023.1324760)
Supplement: Supplementary file 5 [file Presentation_1.pdf]

1 **Supplementary Figure**

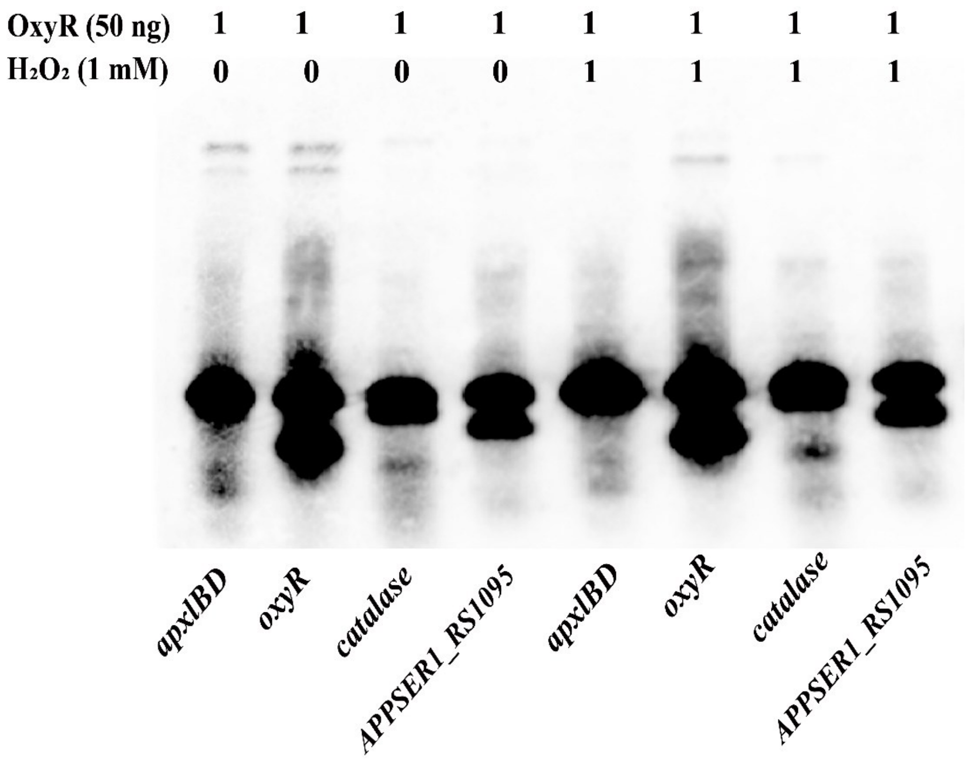

EMSA pre-experiments predicting the binding of OxyR protein to gene promoter sequences. In each reaction system, a total of 50ng of OxyR protein was added to assess its binding to the *apxIBD*, *oxyR*, *catalase*, and *APPSER1_RS1095* gene promoters under conditions with or without the addition of 1mM hydrogen peroxide. The results indicated that OxyR protein could bind to the promoter sequences of *apxIBD*, *oxyR* and *catalase* genes. However, hydrogen peroxide weakened the binding of OxyR protein to these gene promoters.
